# Enrichment and isolation of *Flavobacterium* strains with tolerance to high concentrations of cesium ion

**DOI:** 10.1038/srep20041

**Published:** 2016-02-17

**Authors:** Souichiro Kato, Eri Goya, Michiko Tanaka, Wataru Kitagawa, Yoshitomo Kikuchi, Kozo Asano, Yoichi Kamagata

**Affiliations:** 1 Division of Applied Bioscience, Graduate School of Agriculture, Hokkaido University, Kita-9 Nishi-9, Kita-ku, Sapporo, Hokkaido 060-8589, Japan; 2 Bioproduction Research Institute, National Institute of Advanced Industrial Science and Technology (AIST), 2-17-2-1 Tsukisamu-Higashi, Toyohira-ku, Sapporo, Hokkaido 062-8517, Japan; 3 Research Center for Advanced Science and Technology, The University of Tokyo, 4-6-1 Komaba, Meguro-ku, Tokyo 153-8904, Japan

## Abstract

Interest in the interaction of microorganisms with cesium ions (Cs^+^) has arisen, especially in terms of their potent ability for radiocesium bioaccumulation and their important roles in biogeochemical cycling. Although high concentrations of Cs^+^ display toxic effects on microorganisms, there have been only limited reports for Cs^+^-tolerant microorganisms. Here we report enrichment and isolation of Cs^+^-tolerant microorganisms from soil microbiota. Microbial community analysis revealed that bacteria within the phylum *Bacteroidetes*, especially *Flavobacterium* spp., dominated in enrichment cultures in the medium supplemented with 50 or 200 mM Cs^+^, while *Gammaproteobacteria* was dominant in the control enrichment cultures (in the presence of 50 and 200 mM K^+^ instead of Cs^+^). The dominant *Flavobacterium* sp. was successfully isolated from the enrichment culture and was closely related to *Flavobacterium chungbukense* with 99.5% identity. Growth experiments clearly demonstrated that the isolate has significantly higher tolerance to Cs^+^ compared to its close relatives, suggesting the Cs^+^-tolerance is a specific trait of this strain, but not a universal trait in the genus *Flavobacterium*. Measurement of intracellular K^+^ and Cs^+^ concentrations of the Cs^+^-tolerant isolate and its close relatives suggested that the ability to maintain low intracellular Cs^+^ concentration confers the tolerance against high concentrations of external Cs^+^.

Cesium (Cs) is a Group I alkali metal with atomic number of 55. The physicochemical properties of Cs are highly similar to the other alkali metals, in particular, to potassium (K). Cs generally exists as monovalent cation (Cs^+^) in solution. Special attention has been paid to the radioisotopes of cesium, especially ^134^Cs and ^137^Cs, due to the concern of radioactive pollution arising from nuclear weapon testing and from intentional and unintentional discharge from nuclear power plants, relatively long half-lives, high water solubility, and rapid incorporation into biological systems^1^,^2^.

Although no essential biological function for Cs^+^ has been identified, Cs^+^ transport and accumulation in living organisms, including bacteria, fungi, and plants, are known to date^1^,^2^,^3^,^4^,^5^,^6^,^7^,^8^,^9^,^10^. This feature has offered bioaccumulation as a potential alternative to existing methods for decontamination or recovery of radioactive Cs from environments. In microorganisms and plants, Cs^+^ is principally incorporated via K^+^ transport systems because of its chemical similarity to K^+^ (refs 1, 5). For example, Cs^+^ transport in *Eschericia coli* was shown to occur via a K^+^ uptake system, namely Kup, although the affinity for Cs^+^ is much lower than for K^+^ (ref. 11). Considering the existence of a number of monocation uptake mechanisms, it would not surprising that different microbial species or strains have wide range of ability to transport and accumulate Cs^+^.

High concentration of Cs^+^, generally above the mM order, is toxic to microorganisms^12^,^13^. Two different mechanisms have been inferred as the inhibitory mechanisms of Cs^+^, namely, induction of K^+^ starvation and intracellular toxicity of Cs^+^. K^+^ starvation is induced by an inhibitory effect of Cs^+^ on K^+^-uptake systems and efflux of K^+^ caused by increasing intracellular Cs^+^ concentration^11^,^14^. Intracellular Cs^+^ is also known to compete with K^+^ for essential biochemical functions, including K^+^-dependent enzymes and stabilization of internal structures (e.g., ribosomes), whose activities are restrained by high concentrations of intracellular Cs^+^ (ref. 5). The critical factor of Cs^+^ toxicity toward microorganisms has been speculated as the extracellular [Cs^+^]/[K^+^] ratio rather than absolute concentration of Cs^+^ (ref. 5). Research on microorganisms with tolerance to high concentration of Cs^+^ would offer novel biotechnology, as Cs^+^-tolerant microorganisms are expected to survive under conditions with high intracellular Cs^+^ concentrations and/or harbor Cs^+^-specific transporter systems and Cs^+^-binding proteins. Additionally, the investigation of Cs^+^-tolerant microorganisms is also expected to bring novel insight into the biological significance of Cs^+^. However, there have been only limited reports for Cs^+^-tolerant microorganisms^15^,^16^,^17^, and the mechanisms for Cs^+^-tolerance has not been characterized.

Here we report enrichment and isolation of microorganisms with tolerance to high concentration of Cs^+^. Soil microorganisms were cultivated in the presence of 50 and 200 mM Cs^+^, followed by microbial community analysis based on the sequencing analysis on 16S rRNA gene clone libraries. After enrichment, Cs^+^-tolerant strains, which became dominant in the enrichment cultures, were isolated and subjected to measurement of intracellular Cs^+^/K^+^ concentrations to infer the mechanisms for Cs^+^ tolerance.

## Results and Discussion

### Cs^+^ inhibits growth in *E. coli* and *B. subtilis*

Firstly, inhibitory effects of Cs^+^ were reevaluated by cultivating the representative microorganisms (*E. coli* and *B. subtilis*) in the basal medium supplemented with different concentrations of CsCl (Fig. S1A and B). The growth rates of *E. coli* and *B. subtilis* were nearly halved when supplemented with 50 and 100 mM CsCl, respectively. The growth of both strains was significantly suppressed by the addition of 200 mM CsCl. These results showed that high concentration of Cs^+^ has inhibitory effects on microbial growth. In order to exclude the possibility of the growth inhibition caused by unintentional elevation of ionic strength and/or osmolality by supplementation of CsCl, *E. coli* and *B. subtilis* were cultivated in the medium supplemented with 50 and 200 mM KCl (Fig. S1A and B). The growth of *E. coli* and *B. subtilis* was not suppressed by addition of KCl, suggesting that neither high ionic strength nor high osmolality are the main factors underlying the inhibitory effects of Cs ^+^ on those bacteria.

### Enrichment culture of Cs^+^-tolerant microorganisms

To enrich for Cs^+^-tolerant microorganisms, soil microorganisms were cultivated in medium supplemented with 50 and 200 mM CsCl (designated as 50Cs and 200Cs, respectively), conditions under which the growths of *E. coli* and *B. subtilis* were significantly suppressed. The basal medium itself, and the basal medium supplemented with 50 and 200 mM KCl (designated as BM, 50K, and 200K, respectively) were also used for the enrichment cultures as controls. In the first enrichment cultures, full microbial growth was observed within 2 days for the BM, 50K, 200K cultures, while full growth in the 50Cs and 200Cs cultures required 3 days (data not shown). After the second enrichment, all cultures fully grew within one day of incubation. Also the O_2_ consumption and the final OD_600_ values were similar within all enrichment cultures. These observations suggest that microorganisms with abilities to grow under high concentration of Cs^+^ conditions were successfully enriched.

### *Flavobacterium* spp. dominated in the Cs^+^-supplemented enrichments

**Figure 1:**
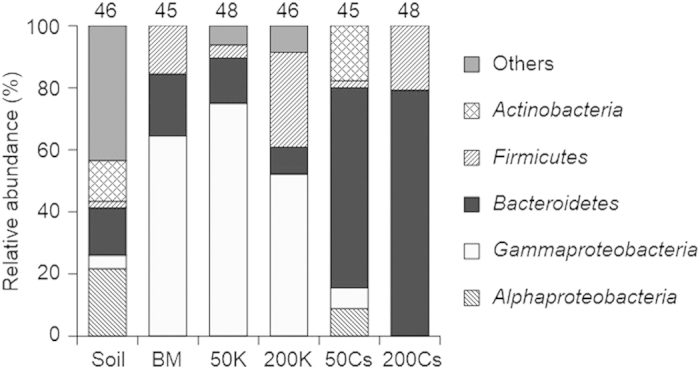
Phylogenetic distribution of bacterial 16S rRNA gene clones in the soil (the inoculum) and enrichment cultures. BM; enriched in the basal medium, 50K and 200K; enriched in the basal medium with 50 and 200 mM KCl, 50Cs and 200Cs; enriched in the basal medium with 50 and 200 mM CsCl. The number above each bar indicates the total number of sequenced clones.

Clone library analysis of 16S rRNA gene was conducted to identify which kind of microorganisms selectively grew in each enrichment culture. Genomic DNA isolated from the forest soil (used as the inoculum) and the early stationary phase of fourth enrichment cultures was subjected to PCR amplification of partial 16S rRNA gene, construction of clone library, and sequencing analysis. A phylotype was defined as a unique clone or a group of clones with sequence similarity of >98%. All phylotypes obtained in this study are listed in Table S1. Overall phylogenetic trends shown in Fig. 1 clearly exhibit that the presence of Cs^+^, but not K^+^, strongly affects the microbial compositions in the enrichment cultures.

Similar phylogenetic distribution patterns were observed for the BM, 50K, and 200K enrichments: clones classified into class *Gammaproteobacteria* shared large fractions (52 to 75% of the total clones). Most of the *Gammaproteobacteria* phylotypes dominated in the BM, 50K, and 200K enrichments are closely related to *Pseudomonas* sp., *Acinetobacter* sp., or *Enterobacter* sp. (Table S1). These microorganisms appear to be susceptible to high concentration of Cs^+^, as few or no *Gammaproteobacteria* phylotypes were retrieved from the 50Cs or 200Cs enrichment, respectively.

**Figure 2:**
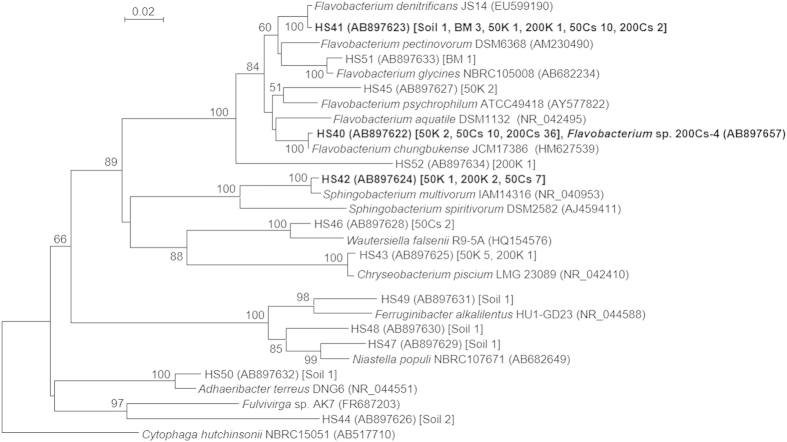
Phylogenetic tree based on partial 16S rRNA gene sequences (approximately 450 bp in length) of clones (classified into phylum *Bacteroidetes*) retrieved in this study and sequences of representative *Bacteroidetes* isolates. Numbers of clones retrieved from different libraries are shown in square brackets. The dominant phylotypes in Cs50 and Cs200 enrichments (HS40, HS41, and HS42) and the isolated strain (*Flavobacterium* sp. 200Cs-4) are highlighted in bold letters. *Cytophaga hutchinsonii* was used as an out-group. Bootstrap values (1,000 trials, only >50% are shown) are indicated at branching points. The bar indicates 2% sequence divergence. Accession numbers of *Bacteroidetes* isolates and clones are shown in parentheses.

The phylogenetic distribution patterns of the 50Cs and 200Cs enrichments were similar to each other and quite distinct from the other enrichments. Most of the clones retrieved from the 50Cs and 200Cs enrichments were classified into phylum *Bacteroidetes* (65 and 79% of the total clones, respectively). While clones classified into *Bacteroidetes* were also retrieved from the inoculum soil and the control enrichments, the relative abundance was not so large (8 to 20% of the total clones). To acquire further phylogenetic information, a phylogenetic tree was constructed based on the sequences of all *Bacteroidetes* phylotypes obtained in this study and representative *Bacteroidetes* isolates (Fig. 2). Three *Bacteroidetes* phylotypes, namely HS40, HS41, and HS42, were dominantly retrieved from the Cs^+^-supplemented enrichments. While these phylotypes were also retrieved from the other enrichment conditions, the abundances were only a minor fraction. The phylotype HS40, closely related to *Flavobacterium chungbukense* CS100 (ref. 18) with 99.0% sequence identity, dominated in both the 50Cs and 200Cs enrichments (22 and 75% of the total clones, respectively), suggesting that this phylotype has high ability to grow under high concentration of Cs^+^. The phylotype HS41, closely related to *Flavobacterium denitrificans* JS14-1 (ref. 19) with 99.6% sequence identity, was also retrieved from both the 50Cs and 200Cs enrichments. While the phylotype HS41 was one of the major phylotypes in the 50Cs enrichment (22% of the total clones), it was minor in the 200Cs enrichment (4.2% of the total clones). The phylotype HS42, closely related to *Sphingobacterium multivorum* IAM14316 (ref. 20) with 98.8% sequence identity, was also one of the major phylotypes in the 50Cs enrichment (16% of the total clones), while it was not retrieved from the 200Cs enrichment. Altogether, the clone library analysis clearly demonstrated that particular *Bacteroidetes* bacteria, especially *Flavobacterium* spp., appear to have significant tolerance to high concentration of Cs^+^.

### Isolation of Cs^+^-tolerant *Flavobacterium* strains

After enrichment, we attempted to isolate Cs^+^-tolerant microorganisms from the 200Cs enrichment culture. The early stationary phase culture of 200Cs enrichment was serially diluted and inoculated onto the gellan gum-solidified basal medium supplemented with 200 mM CsCl. Colonies appeared after 2 to 3 days incubation and were transferred to the same medium for further purification. After the purification, 16 colonies were randomly picked and their partial 16S rRNA gene sequences were determined. Of the isolates, 4 strains had nearly identical sequences (>99.5% sequence identity to each other), and their sequences are closely related to both *F. chungbukense* CS100 and the HS40 phylotype that dominated in the 200Cs enrichment (>99.3% sequence identity). One of the isolates, designated as strain 200Cs-4, was selected as a representative strain for further experiments. The 16S rRNA gene sequence of strain 200Cs-4, containing a continuous stretch of 1448 nt, was determined. The sequence-similarity calculations indicated that the closest relative of strain 200Cs-4 is *F. chungbukense* CS100 (99.5% sequence identity).

### Cs^+^-tolerance of the isolate and its closest relative

**Figure 3:**
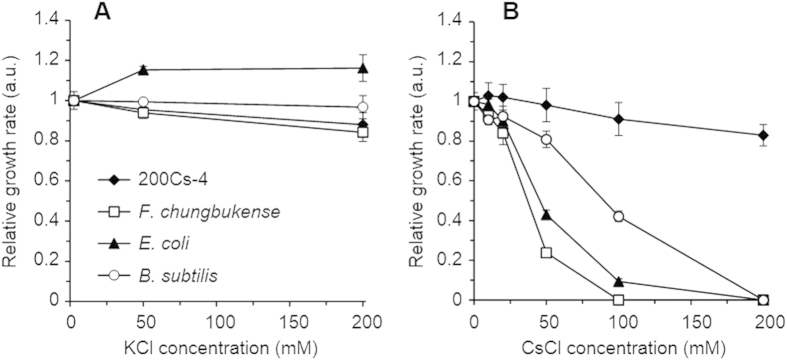
Effects of K^+^and Cs^+^ on the growth of the Cs^+^-tolerant isolate (200Cs-4), the closest relative of the isolate (*F. chungbukense*), and the reference microorganisms (*E. coli* and *B. subtilis*). The relative growth rates of the four strains are plotted against KCl (A) and CsCl (B) concentrations. The growth rates determined from the respective growth curves (Fig. S1) were normalized against those in the basal medium (containing 2.5 mM KCl and no CsCl). Data are presented as the means of three independent cultures, and error bars represent standard deviations.

Strain 200Cs-4 and its closest relative *F. chungbukense* CS100 were cultivated in the basal medium supplemented with different concentrations of CsCl and KCl to evaluate their Cs^+^-tolerance (Fig. S1C and D). The growth rates of strain 200Cs-4, *F. chungbukense* CS100, and the reference microorganisms (*E. coli* and *B. subtilis*) under the Cs^+^- and K^+^-supplemented conditions were compared in Fig. 3. As discussed above, the growth of *E. coli* and *B. subtilis* was severely suppressed by 50 and 100 mM Cs^+^, respectively, and no growth was observed at 200 mM Cs^+^, while 200 mM K^+^ did not have significant effects. The growth rate of *F. chungbukense* CS100 showed similar pattern to that of *E. coli*: 50 mM Cs^+^ decreased the growth rate by 76 ± 2% and no growth was observed at ≥100 mM Cs^+^, while 200 mM K^+^ only slightly decreased the growth rate by 16 ± 4%. On the contrary, strain 200Cs-4 exhibited significant tolerance to Cs^+^. The growth rate of strain 200Cs-4 gradually decreased with increasing Cs^+^ concentration. However, the growth rate was only 17 ± 5% reduced at 200 mM Cs^+^, which is not significantly different from the reduction in the growth rate in 200 mM K^+^ culture (12 ± 6%). These results clearly suggest that *Flavobacterium* sp. strain 200Cs-4 has tolerance to high concentration of Cs^+^, where the ability must have been acquired at the strain level, as it is not a universal feature of phylum *Bacteroidetes* and genus *Flavobacterium*.

### Measurements of intracellular Cs^+^ and K^+^ concentrations

**Figure 4:**
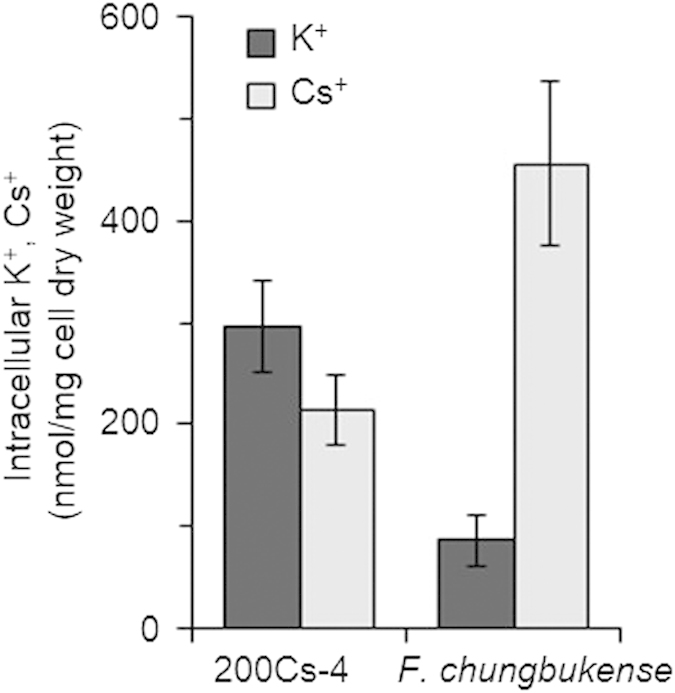
Intracellular K^+^and Cs^+^ concentrations of *Flavobacterium* sp. 200Cs-4 and *F. chungbukense* cultivated in the medium supplemented with 50 mM CsCl. Data are presented as the means of three independent cultures, and error bars represent standard deviations.

Competitive inhibition of K^+^ influx, substitution of intracellular K^+^ with Cs^+^, and intracellular toxicity of Cs^+^ have been supposed to be the mechanisms of Cs^+^ toxicity on microorganisms^5^,^11^,^14^. Considering the inhibitory mechanisms, microbial tolerance to Cs^+^ is supposed to be conferred by ability to maintain low intracellular Cs^+^ and/or tolerance to high intracellular Cs^+^. In order to assess the mechanisms, we determined intracellular K^+^ and Cs^+^ concentrations of Cs^+^-tolerant *Flavobacterium* sp. strain 200Cs-4 and Cs^+^-sensitive *F. chungbukense* CS100 under Cs^+^-stressed conditions (Fig. 4). Cultivation of *F. chungbukense* CS100 with 50 mM of Cs^+^ resulted in much higher intracellular Cs^+^ concentration than K^+^. The ratio of intracellular [Cs^+^]/[K^+^] was 6.2 ± 2.3. This result suggested that incorporation of Cs^+^ that leads to K^+^ release is the causal factor of growth suppression of *F. chungbukense* CS100 as reported for *E. coli*^11^,^14^. On the other hand, intracellular K^+^ and Cs^+^ concentrations of *Flavobacterium* sp. strain 200Cs-4 under the same conditions were significantly higher and lower, respectively, than those of *F. chungbukense* CS100. The ratio of intracellular [Cs^+^]/[K^+^] was 0.72 ± 0.03, which is much smaller than that of *F. chungbukense* CS100. This result indicated that *Flavobacterium* sp. strain 200Cs-4 has ability to maintain low intracellular Cs^+^ concentration, which confers Cs^+^-tolerance on this strain.

K^+^ transport systems with high specificity is one of the plausible mechanisms for maintaining low intracellular Cs^+^. Since Cs^+^ is competitively incorporated into cells through K^+^ transport systems^5^,^11^, microorganisms with K^+^ transporters with high specificity (or in other words, extremely low affinity to Cs^+^) could maintain intracellular Cs^+^ concentration low. Considering that microbial capacity of Cs^+^-uptake considerably differ from species to species^5^, and affinity to Cs^+^ also varies among different K^+^ transporters^11^, it would not be surprising that Cs^+^-tolerant microorganisms harbor special K^+^-transport systems with remarkably high specificity. Incorporation of radiocesium in agricultural plants has been regarded as a serious problem because consumption of food containing radiocesium is the principal route of internal exposure to radiocesium^21^. There are many investigations aiming to decrease transport of Cs^+^ from soils to agricultural plants, including modification of soil properties such as clay content, and K and ammonium statuses^22^,^23^. Further investigations on the highly specific K^+^-transport systems of the Cs^+^-tolerant bacteria will offer new biotechnologies for this research fields, for example, generation of agricultural plants with extremely low radiocesium incorporation by replacing their K^+^ transport systems with those derived from Cs^+^-tolerant microorganisms.

The other putative mechanism for maintaining low intracellular Cs^+^ is specific efflux systems for Cs^+^. In bacteria, efflux systems for metal cations have major roles in tolerance to heavy metals, such as Cu, Cd, and Pb^24^. Also lithium ions (Li^+^), known as a toxic alkali metal cation, is at least partly detoxified by Li^+^ efflux via a proton antiporter in *E. coli*^25^. Considering these findings, it is possible that Cs^+^-tolerant microorganisms have unconventional efflux systems for Cs^+^, while there are so far no reports on microbial Cs^+^ efflux systems. This study will open the possibility of application of Cs^+^-incorporating microorganisms for recovery of radiocesium from environments by introduction of such Cs^+^-specific transport systems into genetically tractable microorganisms.

## Conclusions

Microorganisms with tolerance to high concentration of Cs^+^ were successfully enriched and isolated. The isolate *Flavobacterium* sp. 200Cs-4 vigorously grew in the medium with 200 mM Cs^+^, while its closest relative *F. chungbukense* CS100 did not. This is the first report for isolation of Cs^+^-tolerant microorganisms in the phylum *Bacteroidetes*, while Cs^+^-tolerant microorganisms so far identified had been either *Proteobacteria* or *Actinobacteria*^15^,^16^,^17^. Determination of intracellular K^+^ and Cs^+^ concentrations demonstrated that *Flavobacterium* sp. 200Cs-4 has high ability to maintain low intracellular Cs^+^ concentration under Cs^+^-stressed conditions. Further studies on the newly isolated Cs^+^-tolerant strain will shed light on the novel aspects of interaction between microorganisms and Cs and on development of novel biotechnologies relating to problems of radiocesium contamination.

## Methods

### Bacterial strains and culture conditions

*E. coli* strain K12 (ATCC12435) and *Bacillus subtilis* strain 168 (JCM10629) were routinely cultured at 37 °C in Luria-Bertani medium (pH 7) comprised of 5 g of yeast extract, 10 g of tryptone, and 10 g of NaCl per liter. *Flavobacterium chungbukense* strain CS100 (JCM17386) was regularly cultured at 25 °C in a basal medium consisting of 1.8 g of glucose, 0.3 g of KH_2_PO_4_, 1 g of NH_4_Cl, 0.1 g of MgCl_2_∙6H_2_O, 0.08 g of CaCl_2_∙6H_2_O, 0.6 g of NaCl, 0.02 g of MgSO_4_∙7H_2_O, 9.52 g of 4-(2-hydroxyethyl)-1-piperazineethanesulfonic acid, 0.5 g of yeast extract, and 10 ml each of vitamin solution and trace metal solution per liter of distilled water. The pH of the medium was adjusted to 7.0 by 5N NaOH solution. The vitamin solution contains 2 mg of biotin, 2 mg of folic acid, 10 mg of pyridoxine-HCl, 5 mg of thiamine-HCl, 5 mg of riboflavin, 5 mg of nicotinic acid, 5 mg of Ca-pantothenate, 5 mg of *p*-aminobenzoic acid, 5 mg of lipoic acid, and 0.01 mg of vitamin B12 per liter of distilled water. The trace metal solution contains 12.8 g of nitrilotriacetic acid, 1.35 g of FeCl_3_·6H_2_O, 0.1 g of MnCl_2_·4H_2_O, 0.1 g of CaCl_2_·2H_2_O, 0.1 g of ZnCl_2_, 1 g of NaCl, 0.12 g of NiCl_2_·6H_2_O, 25 mg of CuCl_2_·2H_2_O, 24 mg of CoCl_2_·6H_2_O, 24 mg of Na_2_MoO_4_·2H_2_O, 10 mg of H_3_BO_3_, 4 mg of Na_2_SeO_3_·5H_2_O, and 4 mg of Na_2_WO_4_ per liter of distilled water. Growth experiments of *E. coli*, *B. subtilis*, *F. chungbukense*, and Cs^+^-tolerant isolates were conducted with the basal medium supplemented with different concentrations of KCl and CsCl. All strains were cultivated at 25 °C, except for *E. coli* and *B. subtilis* (at 37 °C), with shaking (130 rpm) aerobically. Growth was monitored by measuring optical density at 600 nm (OD_600_). All culture experiments were conducted in at least triplicate.

### Enrichment cultures

Cs^+^-tolerant microbial communities were enriched in vials (125 ml in capacity) filled with 20 ml of the basal medium supplemented with different concentrations of KCl and CsCl. Approximately 200 mg (wet weight) of soil was inoculated as a source of microorganisms. Soil samples from a depth of 5 to 10 cm below the surface were collected from a deciduous forest in Hokkaido University, Japan. Soil samples were immediately used for cultivation experiments. The vials were sealed with butyl rubber stoppers and aluminum seals, and incubated at 25 °C with shaking (130 rpm). Growth was monitored by measuring the headspace O_2_ concentration with a gas chromatograph (GC-2014, Shimadzu) as described previously^26^. When O_2_ consumption was ceased, 1% (v/v) of enriched culture was transferred to the fresh medium.

### Clone library analysis

Microbial cells for clone library analysis were collected from early stationary phases of the 4th enrichment cultures. Genomic DNA was extracted using the FAST DNA Spin Kit for soil (MP Biomedicals) according to the manufacturer’s instructions. PCR amplification of 16S rRNA gene fragments was performed using a primer pair of 27F (5′- AGA GTT TGA TYM TGG CTC AG -3′) and 533R (5′- TTA CCG CGG CKG CTG RCA C -3′) as described previously^27^. PCR products were purified using a QIAquick PCR Purification Kit (QIAGEN), ligated into pGEM-T Easy Vector (Promega), and cloned into *E. coli* JM109 competent cells (Promega). Sequences of the cloned PCR products were determined at the Dragon Genomics Center (TAKARA Bio).

### Phylogenetic analysis

The rRNA gene sequences with >98% similarity were assigned to the same phylotype using FastGroupII program^28^. The sequence of each phylotype was compared with those stored in the GenBank nucleotide sequence database using the BLAST program^29^. Phylogenetic classification of phylotypes was conducted using the Classifier program in the Ribosomal Database Project database^30^. The sequence of each phylotype was aligned using CLUSTAL W ver. 1.83 (ref. 31), and a phylogenetic tree was constructed by the neighbour-joining method^32^ using program MEGA ver. 5.05 (ref. 33). To evaluate the robustness of an inferred tree, the bootstrap resampling method^34^ was used with 1000 replicates.

### Isolation of Cs^+^-tolerant microorganisms

Cs^+^-tolerant microorganisms were isolated using gellan gum (0.6%)-solidified basal medium supplemented with 200 mM CsCl. Colonies were randomly picked, and further purified with the same medium at least three times. Partial sequences of 16S rRNA gene of isolates were determined as described above. The whole length of 16S rRNA gene sequence was determined by direct sequencing of the DNA fragment PCR-amplified with a primer pair of 27F and 1492R (5′- GGH TAC CTT GTT ACG ACT T -3′) as described previously^35^.

### Determination of intracellular K^+^ and Cs^+^ concentrations

Intracellular K^+^ and Cs^+^ concentrations were determined by the method described by Tomioka *et al.*^6^ with slight modifications. Cells in late-logarithmic growth phases were harvested by centrifugation and washed twice with 0.85% NaCl solution to remove K^+^ and Cs^+^ adsorbed on the cell surfaces. Washed cells were subjected to acid digestion (boiled in 1% HCl solution for 10 min), followed by dilution with 1% HCl solution with 1 g l^−1^ of either KCl (for Cs^+^ measurements) or CsCl (for K^+^ measurements). The K^+^ and Cs^+^ contents in the suspension were determined by an atomic absorption spectrophotometer Z-5310 (Hitachi Kyowa Engineering).

### Nucleotide sequence accession numbers

The nucleotide sequence data reported here have been submitted to GenBank under Accession No. AB897583-AB897657.

## Additional Information

**How to cite this article**: Kato, S. *et al.* Enrichment and isolation of *Flavobacterium* strains with tolerance to high concentrations of cesium ion. *Sci. Rep.* **6**, 20041; doi: 10.1038/srep20041 (2016).

## Supplementary Material

Supplementary Information

## References

[b1] White P. J. & Broadley M. R. Mechanisms of caesium uptake by plants. New Phytol. 147, 241–256 (2000).

[b2] Zhu Y. G. & Shaw G. Soil contamination with radionuclides and potential remediation. Chemosphere 41, 121–128 (2000). 10819188 10.1016/s0045-6535(99)00398-7

[b3] Avery S. V., Codd G. A. & Gadd G. M. Caesium accumulation and interactions with other monovalent cations in the cyanobacterium *Synechocystis* PCC 6803. J. Gen. Microbiol. 137, 405–413 (1991).

[b4] Avery S. V., Codd G. A. & Gadd G. M. Replacement of cellular potassium by caesium in *Chlorella emersonii*: differential sensitivity of photoautotrophic and chemoheterotrophic growth. J. Gen. Microbiol. 138, 69–76 (1992).

[b5] Avery S. V. Caesium accumulation by microorganisms: uptake mechanisms, cation competition, compartmentalization and toxicity. J. Ind. Microbiol. 14, 76–84 (1995). 7766213 10.1007/BF01569888

[b6] Tomioka N., Uchiyama H. & Yagi O. Isolation and characterization of cesium-accumulating bacteria. Appl. Environ. Microbiol. 58, 1019–1023 (1992). 1575473 10.1128/aem.58.3.1019-1023.1992 PMC195371

[b7] Tomioka N., Uchiyama H. & Yagi O. Cesium accumulation and growth characteristics of *Rhodococcus erythropolis* CS98 and *Rhodococcus* sp. strain CS402. Appl. Environ. Microbiol. 60, 2227–2231 (1994). 16349312 10.1128/aem.60.7.2227-2231.1994 PMC201636

[b8] Kuwahara C. *et al.* Studies on uptake of cesium by mycelium of the mushroom (*Pleurotus ostreatus*) by ^133^Cs-NMR. J. Radioanal. Nucl. Chem. 235, 191–194 (1998).

[b9] Gyuricza V., Declerck S. & Dupré de Boulois H. Arbuscular mycorrhizal fungi decrease radiocesium accumulation in *Medicago truncatula*. J. Environ. Radioact. 101, 591–596 (20109. 20378216 10.1016/j.jenvrad.2010.03.004

[b10] Kuwahara C. *et al.* Characteristics of cesium accumulation in the filamentous soil bacterium *Streptomyces* sp. K202. J. Environ. Radioact. 102, 138–144 (2011). 21163559 10.1016/j.jenvrad.2010.11.004

[b11] Bossemeyer D., Schlösser A. & Bakker E. P. Specific cesium transport via the *Escherichia coli* Kup (TrkD) K^+^ uptake system. J. Bacteriol. 171, 2219–2221 (1989). 2649491 10.1128/jb.171.4.2219-2221.1989 PMC209881

[b12] Perkins J. & Gadd G. M. The influence of pH and external K^+^ concentration on caesium toxicity and accumulation in *Escherichia coli* and *Bacillus subtilis*. J. Ind. Microbiol. 14, 218–225 (1995). 7598839 10.1007/BF01569931

[b13] Hampton C. R. *et al.* Cesium toxicity in *Arabidopsis*. Plant Physiol. 136, 3824–3837 (2004). 15489280 10.1104/pp.104.046672 PMC527179

[b14] Jung K., Krabusch M. & Altendorf K. Cs^+^ induces the *kdp* operon of *Escherichia coli* by lowering the intracellular K^+^ concentration. J. Bacteriol. 183, 3800–3803 (2001). 11371546 10.1128/JB.183.12.3800-3803.2001 PMC95259

[b15] Kato F. *et al.* Accumulation and subcellular localization of cesium in mycelia of *Streptomyces lividans* and a Cs tolerant strain, *Streptomyces* sp. TOHO-2. J. Health Sci. 46, 259–262 (2000).

[b16] Monsieurs P. *et al.* Heavy metal resistance in *Cupriavidus metallidurans* CH34 is governed by an intricate transcriptional network. Biometals. 24, 1133–1151 (2011). 21706166 10.1007/s10534-011-9473-y

[b17] Dekker L., Osborne T. H. & Santini J. M. Isolation and identification of cobalt and caesium resistant bacteria from a nuclear fuel storage pond. FEMS Microbiol. Lett. 359, 81–84 (2014). 25091383 10.1111/1574-6968.12562

[b18] Lim C. S. *et al.* *Flavobacterium chungbukense* sp. nov., isolated from soil. Int. J. Syst. Evol. Microbiol. 61, 2734–2739 (2011). 21186289 10.1099/ijs.0.028563-0

[b19] Horn M. A. *et al.* *Dechloromonas denitrificans* sp. nov., *Flavobacterium denitrificans* sp. nov., *Paenibacillus anaericanus* sp. nov. and *Paenibacillus terrae* strain MH72, N_2_O-producing bacteria isolated from the gut of the earthworm *Aporrectodea caliginosa*. Int. J. Syst. Evol. Microbiol. 55, 1255–1265 (2005). 15879265 10.1099/ijs.0.63484-0

[b20] Yabuuchi E. *et al.* *Sphingobacterium* gen. nov., *Sphingobacterium spiritivorum* comb. nov., *Sphingobacterium multivorum* comb. nov., *Sphingobacterium mizutae* sp. nov., and *Flavobacterium indologenes* sp. nov.: glucose-nonfermenting gram-negative rods in CDC groups IIK-2 and IIb. Int. J. Syst. Bacteriol. 33, 580–598 (1983).

[b21] Zhu Y. G. & Smolders E. Plant uptake of radiocaesium: a review of mechanisms, regulation and application. J. Exp. Bot. 51, 1635–1645 (2000). 11053452 10.1093/jexbot/51.351.1635

[b22] Absalom J. P. *et al.* Predicting soil to plant transfer of radiocesium using soil characteristics. Environ. Sci. Technol. 33, 1218–1223 (1999).

[b23] Absalom J. P. *et al.* Predicting the transfer of radiocaesium from organic soils to plants using soil characteristics. J. Environ. Radioact. 52, 31–43 (2001). 11202684 10.1016/s0265-931x(00)00098-9

[b24] Nies D. H. Efflux-mediated heavy metal resistance in prokaryotes. FEMS Microbiol. Rev. 27, 313–339 (2003). 12829273 10.1016/S0168-6445(03)00048-2

[b25] Inaba K. *et al.* Lithium toxicity and Na^+^ (Li^+^)/H^+^antiporter in *Escherichia coli*. Biol. Pharm. Bull. 17, 395–398 (1994). 8019504 10.1248/bpb.17.395

[b26] Kato S. *et al.* Physiological and transcriptomic analyses of the thermophilic, aceticlastic methanogen *Methanosaeta thermophila* responding to ammonia stress. Microbes Environ. 29, 162–167 (2014). 24920170 10.1264/jsme2.ME14021 PMC4103522

[b27] Kato S. *et al.* Respiratory interactions of soil bacteria with (semi)conductive iron-oxide minerals. Environ. Microbiol. 12, 3114–3123 (2010). 20561016 10.1111/j.1462-2920.2010.02284.x

[b28] Yu Y., Breitbart M., McNairnie P. & Rohwer F. FastGroupII: a web-based bioinformatics platform for analyses of large 16S rDNA libraries. BMC Bioinformatics 7, 57 (2006). 16464253 10.1186/1471-2105-7-57 PMC1386709

[b29] Altschul S. F. *et al.* Basic local alignment search tool. J. Mol. Biol. 215, 403–410 (1990). 2231712 10.1016/S0022-2836(05)80360-2

[b30] Wang Q., Garrity G. M., Tiedje J. M. & Cole J. R. Naive Bayesian classifier for rapid assignment of rRNA sequences into the new bacterial taxonomy. Appl. Environ. Microbiol. 73, 5261–5267 (2007). 17586664 10.1128/AEM.00062-07 PMC1950982

[b31] Thompson J. D., Higgins D. G. & Gibson T. J. CLUSTAL W: improving the sensitivity of progressive multiple sequences alignment through sequence weighting, position-specific gap penalties and weight matrix choice. Nucleic Acids Res. 22, 4673–4680 (1994). 7984417 10.1093/nar/22.22.4673 PMC308517

[b32] Saitou N. & Nei M. The neighbor-joining method: a new method for reconstructing phylogenetic trees. Mol. Biol. Evol. 4, 406–425 (1987). 3447015 10.1093/oxfordjournals.molbev.a040454

[b33] Tamura K., Dudley J., Nei M. & Kumar S. MEGA4: Molecular Evolutionary Genetics Analysis (MEGA) software version 4.0. Mol. Biol. Evol. 24, 1596–1599 (2007). 17488738 10.1093/molbev/msm092

[b34] Felsenstein J. Confidence limits on phylogenies: an approach using the bootstrap. Evolution 39, 783–791 (1985). 10.1111/j.1558-5646.1985.tb00420.x 28561359

[b35] Kato S. *et al.* *Clostridium straminisolvens* sp. nov., a moderately thermophilic, aerotolerant and cellulolytic bacterium isolated from a cellulose-degrading bacterial community. Int. J. Syst. Evol. Microbiol. 54, 2043–2047 (2004). 15545431 10.1099/ijs.0.63148-0

