# Supplementary material for: Enrichment and isolation of Flavobacterium strains with tolerance to high concentrations of cesium ion
Source: Sci Rep. 2016 Feb 17;6:20041. doi: 10.1038/srep20041 (PMC4756683; doi:10.1038/srep20041)
Supplement: Supplementary Information [file srep20041-s1.pdf]

Enrichment and isolation of *Flavobacterium* strains with tolerance to high concentrations of cesium ion

Authors:

Souichiro Kato[1,2,3*], Eri Goya[1], Michiko Tanaka[1], Wataru Kitagawa[1,2], Yoshitomo Kikuchi[1,2], Kozo Asano[1], Yoichi Kamagata[1,2]

[1]*Division of Applied Bioscience, Graduate School of Agriculture, Hokkaido University, Kita-9 Nishi-9, Kita-ku, Sapporo, Hokkaido 060-8589, Japan*

[2]*Bioproduction Research Institute, National Institute of Advanced Industrial Science and Technology (AIST), 2-17-2-1 Tsukisamu-Higashi, Toyohira-ku, Sapporo, Hokkaido 062-8517, Japan*

[3]*Research Center for Advanced Science and Technology, The University of Tokyo, 4-6-1 Komaba, Meguro-ku, Tokyo 153-8904, Japan*

*For correspondence. E-mail s.katou@aist.go.jp; Tel (+81) 11 857 8968; Fax (+81) 11 857 8915

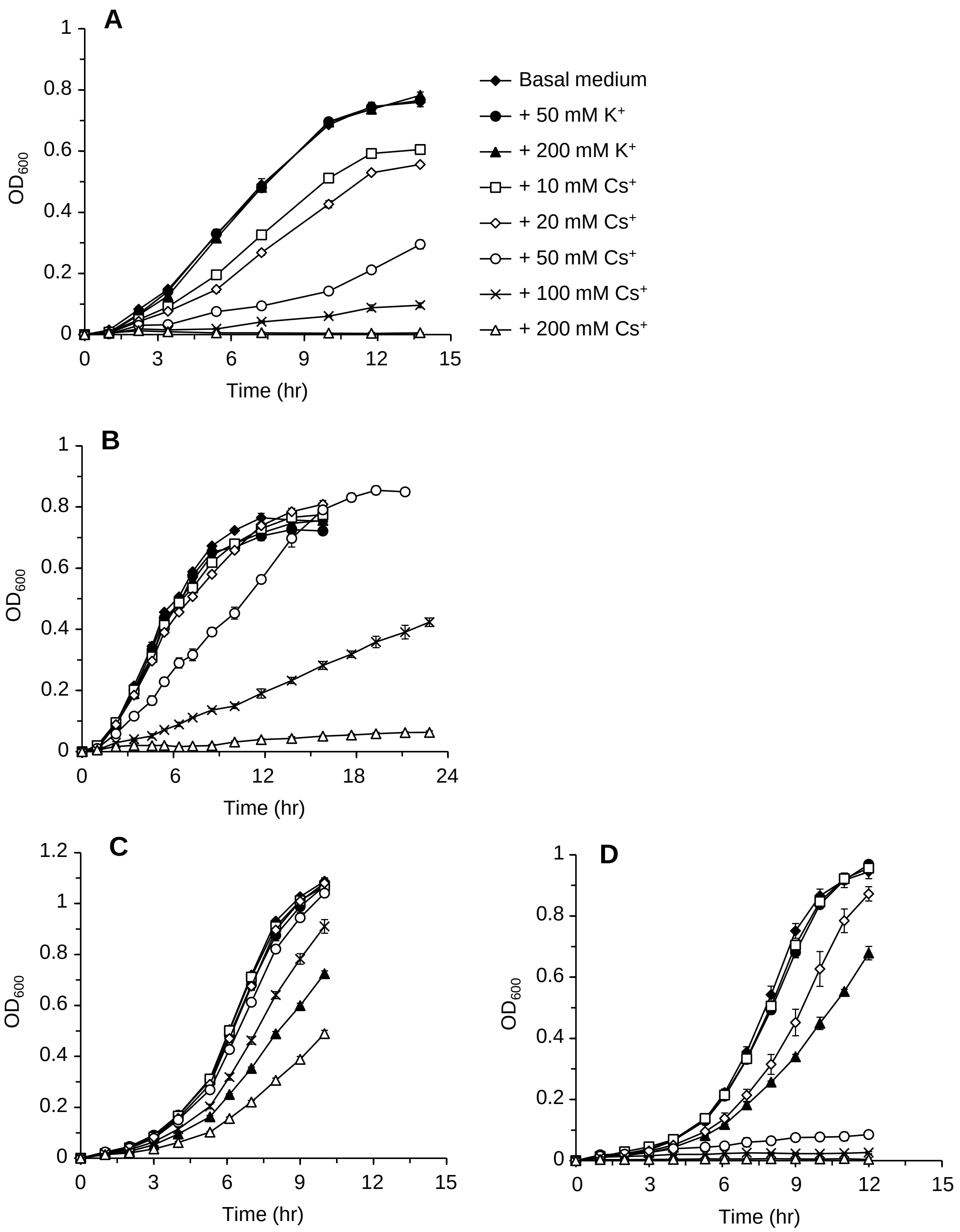

FIG S1 Growth curves of (A) *E. coli*, (B) *B. subtilis*, (C) the Cs^+-tolerant isolate (strain 200Cs-4) and (D) the closest relative of the isolate (*F. chungbukense* CS100) with different concentrations of KCl and CsCl as described in the figure. Data are presented as means of three independent cultures, and error bars represent standard deviations.

Table S1 The phylotypes detected in the soil and the enrichment cultures.

Phylogenetic group	Phylotype	Closest relative (Similarity, %)	Number of clones					
			Soil	FB	50K	200K	50Cs	200Cs
Alphaproteobacteria	HS01	AB121773 *Bradyrhizobium* sp. Shinshu-th2 (94)	4					
	HS02	FJ859687 *Ochrobactrum pseudogrignonense* BIHB 340 (99)					4	
	HS03	AF395032 *Sphingomonas* sp. SIA181-1A1 (99)	2					
	HS04	GQ369056 *Azospirillum* sp. T2-YC6788 (96)	1					
	HS05	GU269549 *Pedomicrobium manganicum* ATCC 33121 (96)	1					
	HS06	AJ563927 *Methylocella palustris* H4 (91)	1					
	HS07	AM412118 *Chelatococcus* sp. P-117 (94)	1					
Betaproteobacteria	HS08	JN674090 *Comamonas* sp. p19 (100)			3	2		
	HS09	HM044161 *Ferrovum myxofaciens* P3G (90)	1					
	HS10	AB649026 *Variovorax ginsengisoli* S32319 (96)	1					
	HS11	HQ005420 *Variovorax paradoxus* HB44 (98)	1					
	HS12	AJ551104 *Burkholderia* sp. wp26 (93)				1		
	HS13	NR_028719 *Comamonas terrigena* IMI 359870 (99)				1		
Gammaproteobacteria	HS14	KC195874 *Pseudomonas putida* BF111 (99)		4	18	2		
	HS15	EU337121 *Acinetobacter johnsonii* 3B2 (99)		3		11		
	HS16	DQ133596 *Pantoea agglomerans* B1 (99)		5	5	2		
	HS17	HM854373 *Enterobacter cloacae* P04 (99)		5	1	5		
	HS18	HM231274 *Raoultella ornithinolytica* NB1 (99)		2	3	1		
	HS19	EF520801 *Erwinia* sp. AUEY28 (98)		2	1	1		
	HS20	AF506040 *Pseudomonas fluorescens* LCSA0TU1 (99)		2	1			
	HS21	X60411 *Aeromonas eucrenophila* NCIMB 74 (99)		1			2	
	HS22	JQ267508 *Enterobacter aerogenes* ger-BHI10 (99)			3			
	HS23	FM164626 *Pseudomonas* sp. PC IW 25 (98)			2			

Table S1. Continued.

Phylogenetic group	Phylotype	Closest relative (Similarity, %)	Number of clones					
			Soil	FB	50K	200K	50Cs	200Cs
Gammaproteobacteria	HS24	GU586313 *Stenotrophomonas rhizophila* IR (99)			2			
	HS25	AY234531 Bacterium Ellin5114 (94)	1					
	HS26	FN554396 Gamma proteobacterium CH43 (91)	1					
	HS27	FJ940849 *Aeromonas veronii* CYJ205 (97)		1				
	HS28	HQ132731 *Leclercia adecarboxylata* Ld1 (98)		1				
	HS29	JQ954967 *Serratia* sp. ZJ-2 (96)		1				
	HS30	EU304285 *Pseudomonas* sp. 73 (92)		1				
	HS31	FR687005 *Aeromonas hydrophila* Pa031 (96)		1				
	HS32	JX899634 *Pseudomonas* sp. REp-amp_124 (95)				1		
	HS33	HQ407278 *Enterobacter cancerogenus* H103 (99)				1		
	HS43	KC213922 *Leucobacter komagatae* Z3_S (97)				1		
	HS34	HQ436039 *Aeromonas* sp. MDC2473 (96)					1	
Deltaproteobacteria	HS35	JQ346737 *Syntrophus gentianae* HQGOe1 (86)	2					
	HS36	AJ233940 *Chondromyces pediculatus* Cm p17 (85)	1					
	HS37	GU249611 *Sorangiineae bacterium* SBSr004 (82)	1					
	HS38	CP002297 *Desulfovibrio vulgaris* RCH1 (81)	1					
	HS39	HF543825 *Kofleria flava* DSM14620 (87)	1					

Table S1. Continued.

Phylogenetic group	Phylotype	Closest relative (Similarity, %)	Number of clones					
			Soil	FB	50K	200K	50Cs	200Cs
Bacteroidetes	HS40	HM627539 *Flavobacterium chungbukense* CS100 (99)			2		10	36
	HS41	HQ882702 *Flavobacterium denitrificans* JS14-1 (99)	1	3	1	1	10	2
	HS42	NR_040953 *Sphingobacterium multivorum* IAM14316 (99)			1	2	7	
	HS43	AY468484 *Chryseobacterium* sp. LDVH 3 (99)		5	1			
	HS44	AB308367 Bacterium TG141 (91)	2					
	HS45	AY436806 *Bacteroidetes* bacterium RD4.3 (99)			2			
	HS46	HQ154576 *Wautersiella falsenii* R9-5A (96)					2	
	HS47	EU917053 *Niastella* sp. RHYL-67 (96)	1					
	HS48	AM411962 *Sphingobacterium* sp. P-38 (95)	1					
	HS49	AB308369 Bacterium TG124 (95)	1					
	HS50	EF647593 *Adhaeribacter* sp. KS35 (99)	1					
	HS51	AB682234 *Flavobacterium glycines* NBRC 105008 (99)		1				
	HS52	AY538269 *Bacteroidetes* bacterium M2 (97)				1		
Firmicutes	HS53	JN020634 *Bacillus thuringiensis* (99)				10		7
	HS54	FJ970034 *Exiguobacterium acetylicum* QD-3 (99)		3	2			
	HS55	JQ943914 *Lysinibacillus fusiformis* H5 (99)		1		3		
	HS56	AB376084 *Paenibacillus* sp. YT0147 (83)	1					
	HS57	EF101552 *Bacillus psychrodurans* KOPRI24934 (98)					1	

Table S1. Continued.

Phylogenetic group	Phylotype	Closest relative (Similarity, %)	Number of clones					
			Soil	FB	50K	200K	50Cs	200Cs
Actinobacteria	HS58	AY211130 *Arthrobacter protophormiae* Mali 36 (98)					8	
	HS59	AB021325 Bacterium rJ7 (93)	1					
	HS60	AY673309 *Acidimicrobidae* bacterium Ellin7143 (91)	1					
	HS61	NR_041871 *Nocardia cummidelens* DSM 44490 (99)	1					
	HS62	JX949768 *Compostimonas* sp. MDB2-25 (97)	1					
	HS63	JF423906 *Gaiella occulta* F2-233 (90)	1					
	HS64	HM748674 Bacterium Ellin6526 (98)	1					
Acidobacteria	HS65	JF707525 *Acidobacteria* bacterium UCL-085 (91)	2					
	HS66	HM748653 Bacterium Ellin6505 (94)	1					
	HS67	HM748677 Bacterium Ellin6529 (94)	1					
	HS68	DQ075304 Bacterium Ellin640 (95)	1					
Gemmatimonadetes	HS69	HM154525 *Gemmatimonadetes* bacterium KBS708 (85)	1					
	HS70	NR_074708 *Gemmatimonas aurantiaca* T-27 (84)	1					
Verrucomicrobia	HS71	FJ405899 *Verrucomicrobia* bacterium WSF2-44 (90)	1					
	HS72	AB245341 *Spartobacteria* bacterium Gsoil 133 (93)	1					
Chloroflexi	HS73	NR_074379 *Sphaerobacter thermophilus* DSM 20745 (83)	1					
Planctomycetes	HS74	FJ405890 *Planctomycetacia* bacterium WSF3-27 (86)	1					
		Total	46	45	48	46	45	48
